# Commentary: Empathy and its discontents

**DOI:** 10.3389/fpsyg.2017.00542

**Published:** 2017-04-18

**Authors:** Daniel Västfjäll, Arvid Erlandsson, Paul Slovic, Gustav Tinghög

**Affiliations:** ^1^Judgment, Emotion, Decision, Intuition Lab, Linköping UniversityLinköping, Sweden; ^2^Decision ResearchEugene, OR, USA; ^3^Department of Psychology, Lund UniversityLund, Sweden; ^4^Department of Psychology, University of OregonEugene, OR, USA

**Keywords:** empathy, compassion, decision making, psychic numbing, public policy

## We should be equally discontent with empathy and compassion

In “Empathy and its discontents” Bloom (2017: see also Bloom, 2016) argues that we should abandon empathy as a moral compass in favor of compassion. Bloom's central premise is that empathy is narrow in its focus on single identified individuals, biased in that it favors the in-group, and can be used as a tool to motivate us to do things that are not optimally effective, or even destructive (e.g., motivate war). For all these reasons, Bloom argues that policy decision should not be motivated by empathy. There is indeed ample evidence that empathy is fraught with biases and we have, as Bloom, argued that deliberate mechanisms are needed to counteract the innumeracy and parochialism of empathy (Slovic and Västfjäll, 2010). While there is much to agree with Bloom on, there are a few points where we disagree; (1) the definition of compassion, (2) data supporting why empathy, but not compassion, is bad, (3) the role of deliberation in moral judgment.

## Well-defined “empathy” but Ill-defined “compassion”

Bloom is against empathy but for compassion. Whereas, empathy in Bloom's definition is clearly explained and consistent in different papers (“experiencing what you think others are experiencing”), the same cannot be said for compassion. In the Trend in Cognitive Sciences article [1] it is “positive feelings toward others, a desire that others do well and do not suffer…”, but elsewhere it is defined by Bloom similarly as to what Batson (2011) refers to as empathic concern (e.g., “*I am often touched by things that I see happen*”; (Jordan et al., 2016), or as “*a more distanced love and kindness and concern for others* ” (Bloom, 2014). Adding to the confusion, other compassion scholars define it as “*feeling sorrow or concern for the suffering of another person, coupled with the desire to alleviate that suffering*” (Keltner, 2014). This conceptual vagueness makes it difficult to tell how compassion may be “*better prod to moral action*” (Bloom, 2017, p. 24) and to make such a claim we need to carefully distinguish how compassion is different from empathy.

## Data supporting why empathy is bad

It could be argued that no matter which definition of compassion one prefer, relying on it as a moral motive will lead to just as serious biases as relying on empathy. In fact, much of the literature Bloom cites to support his argument does not assess empathy (as he defines it) but is more in line with his definition of compassion. For instance, the work cited by Bloom on the identifiable victim effect (giving more to a victim with a name and a face compared to a statistical victim; Slovic, 2007) as well as the singularity effect (giving more to the one than to the many) has not been found to be driven by empathy but rather a drop in compassion (as measured both by self-report and physiology; Västfjäll et al., 2014). Compassion scholars acknowledge the biased nature of compassion: “…*individuals who encounter the suffering of large numbers of people (e.g. starvation in Sudan) are more likely to respond with sadness, distress or even apathy as a result of their inability to cope with suffering of such a large magnitude*” (Keltner, 2014, p. 331). In support of his distinction between empathy and compassion, Bloom cites work by Singer and Klimecki (2014) on the difference between mindul cold compassion and empathic affect sharing. Although we recognize that compassion training could lead to different brain activity and a smaller risk of apathy than empathy training in some cases, this does not imply that compassion is not fraught with many of the same biases as empathy. Given this it is difficult for us to see how compassion could be a better moral compass than empathy. We think of them as equally poor without the guidance of deliberative analysis.

## The role of reason

One reading of Bloom is that he is not against empathy *per se*, but rather, against emotions in general (including empathy but also compassion, kindness, and love) as the motivator of moral decisions (see also commentary by Zaki, 2017). Bloom argues that utilitarian cost-benefit analyses and moral principles would be better guides for moral behavior. We agree that these are good alternatives for deciding what to do and what to avoid. However, it would not be fair to blame all biases on emotions. Previous work shows that biased cost-benefit analyses underlie the proportion dominance effect (saving 20 out of 25 rather than 22 out of 300), and perceived responsibility underlies the ingroup effect (helping few fellow countrymen rather than many from another country; Fetherstonhaugh et al., 1997; Erlandsson et al., 2015). Further, there is evidence that moral decisions made under intuitive modes of thinking are no different from those that are made deliberatively Tinghög et al., 2016). Although we believe that both empathy and compassion are poor guides when assessing what is right and wrong, emotional reactions can be useful tools for motivating people to act morally. For example, highly emotional information in the form of personal stories or dramatic images (such as the picture of Alan Kurdi—the dead Syrian boy on a beach in Turkey) is often the spark that motivates people to act (Slovic et al., 2017). In these situations, intense emotions can motivate people to e.g. start making monthly donations, which likely will continue long after the emotional reactions have disappeared, thus benefitting also statistical victims. Another related point is brought up by Zaki (2017)—affect and deliberation are intertwined and completely separating reason from feelings in moral decisions is likely an impossible task.

In sum, we agree with Bloom that people rely too single-mindedly on emotions (both empathy and compassion) when making moral decisions. However, some form of emotion (regardless of how biased it is) may be a necessary, though not sufficient, condition for action by both individuals and societies. Zaki (2017) points to the fact that emotion are not inherently good or bad—rather it is the context that determine their functional value. We agree that this is especially true for the affective component of compassion and empathy—sometimes emotion is needed to energize action, sometimes emotion will lead to biased decisions. What we need are deliberatively designed policies, laws, and institutions that can capitalize on our emotional responses and transform their motivational push to align with our moral values consistently across contexts (Slovic, 2007; Slovic et al., 2017).

## Author contributions

All authors listed, have made substantial, direct and intellectual contribution to the work, and approved it for publication.

## Funding

This research was funded by grants from National Science Foundation, Vetenskapsrådet, and Riksbankens Jubileumsfond.

### Conflict of interest statement

The authors declare that the research was conducted in the absence of any commercial or financial relationships that could be construed as a potential conflict of interest.

## References

[B1] Batson C. D. (2011). Altruism in Humans. New York, NY: Oxford University Press.

[B2] Bloom P. (2014). Against Empathy. Boston Review. Available online at: http://bostonreview.net/forum/paul-bloom-against-empathy

[B3] Bloom P. (2016). Against Empathy: The Case of Rational Compassion. New York, NY: Ecco Press.

[B4] Bloom P. (2017). Empathy and its discontents. Trends Cogn. Sci. 21, 24–31. 10.1016/j.tics.2016.11.004 27916513

[B5] Erlandsson A. Björklund F. Bäckström M. (2015). Emotional reactions, perceived impact and perceived responsibility mediate the identifiable victim effect, proportion dominance effect and in-group effect respectively. Organ. Behav. Hum. Decis. Process. 127, 1–14. 10.1016/j.obhdp.2014.11.003

[B6] Fetherstonhaugh D. Slovic P. Johnson S. M. Friedrich J. (1997). Insensitivity to the value of human life: a study of psychophysical numbing. J. Risk Uncertain. 14, 283–300.

[B7] Jordan M. R. Amirm D. Bloom P. (2016). Are empathy and concern psychologically distinct?. Emotion 16:1107. 10.1037/emo0000228 27668802

[B8] Keltner D. (2014). Compassion. H Pos Emo. New York, NY: Guilford.

[B9] Singer T. Klimecki O. M. (2014). Empathy and compassion. Curr. Biol. 24, R875–R878. 10.1016/j.cub.2014.06.054 25247366

[B10] Slovic P. (2007). “If I look at the mass I will never act”: psychic numbing and genocide. Judgm. Decis. Mak. 2, 79–95.

[B11] Slovic P. Västfjäll D. (2010). Affect, moral intuition, and risk. Psychol. Inquiry 21, 387–398. 10.1080/1047840X.2010.521119

[B12] Slovic P. Västfjäll D. Erlandsson A. Gregory R. (2017). Iconic photographs and the ebb and flow of empathic response to humanitarian disasters. Proc. Natl. Acad. Sci. U.S.A. 114, 640–644. 10.1073/pnas.1613977114 28074038 PMC5278443

[B13] Tinghög G. Andersson D. Bonn C. Johannesson M. Kirchler M. Koppel L.. (2016). Intuition and moral decision-making – the effect of time pressure and cognitive load on moral judgment and altruistic behavior. PLoS ONE 11:e0164012. 10.1371/journal.pone.0164012 27783704 PMC5082681

[B14] Västfjäll D. Slovic P. Mayorga M. Peters E. (2014). Compassion fade: affect and charity are greatest for a single child in need. PLoS ONE 9:e100115. 10.1371/journal.pone.0100115 24940738 PMC4062481

[B15] Zaki J. (2017). Moving beyond sterotypes of empathy. Trends Cogn. Sci. 21, 59–60. 10.1016/j.tics.2016.12.004 28040334

